# Racism against Totonaco women in Veracruz: Intercultural competences for health professionals are necessary

**DOI:** 10.1371/journal.pone.0227149

**Published:** 2020-01-14

**Authors:** Niels Michael Dörr, Gunther Dietz

**Affiliations:** 1 Instituto de Investigaciones en Educación, Universidad Veracruzana, Xalapa, Veracruz, Mexico; 2 Institute of History of Medicine of the Justus Liebig University in Giessen, Giessen, Germany; Graduate School of Public Health and Health Policy, City University of New York, UNITED STATES

## Abstract

Racism is a neglected but relevant cause of health disparities within multi-ethnic societies. Different types of racism and other expressions of discrimination must be recognized, critically analyzed, and actively reverted. This paper is based on anthropological fieldwork conducted in three medical facilities in the indigenous region Sierra de Totonacapan in the highlands of Veracruz in Mexico and analyzes maternal health and identifies levels of racism as perceived by female indigenous patients. Applying a theoretical framework that defines racism at three levels, namely, institutionalized, personally mediated, and internalized racism. We empirically distinguish and acknowledge human rights omissions and violations and then analyze the sources of racism in close relation to an intersectional view on gender-, class-, and race-based forms of discrimination. Finally, in addition to investment in health goods and skilled birth attendants, we propose an intercultural competence approach to manage racism, among other ideologies. This approach targets health professionals as conscious, reflexive, and transformative actors of intercultural interactions with culturally diverse patients.

## Introduction

Most societies are racist, and this phenomenon results in health inequity. Nowadays, racism is identified as a relevant but neglected and often ignored health concern [1, 2, 3, 4]. Previous research in multi-ethnic countries in the global North like the United States [5], the United Kingdom [6], Australia [7] and New Zealand [8], but also in multi-ethnic countries in the South such as Brazil [9], Mexico [10] and comparatively Brazil, Mexico, Colombia and Peru [11] emphasized racism as the cause of persistent health disparities. For example, US-based research on health disparities by race has provided evidence of significant inequities between the nation’s African American and white population. In the United States, African Americans have higher death rates than their white counterparts for most leading causes of death [12]. However, health disparities are not biologically or culturally determined; they are explained by a complex structure of social, economic, political, and other factors [13]. Therefore, health disparities related to racism are a crucial argument for the significance of social determinants of health [14, 15]. Regarding human rights, the neglect of racism and other discriminations are a considerable omission. Notably, the accessibility to health facilities, goods, and services without discrimination is fundamental [16].

In Mexico, anthropologists have emphasized that racism is a neglected health concern. Among others, the work of the Mexican anthropologist Menéndez suggested that daily racism is a public health omission in Mexico [17, 18]. In 2003, the Pan American Health Organization confirmed in the Latin American region that *“ethnic origin has a significant impact on health [*…*]*, *namely*: *differentials in health status and life expectancy at birth; differential access to health care*, *disease prevention*, *and health promotion services; differentials in the attention received from health care providers; and differentials in the quality of services”* [19].

Roldán et al., in their research on marginalization and health service coverage, identified a significant shortage in relation to indigenous ethnicity. The authors argued that indigenous groups represent the extreme end of marginalization and access to medical coverage in Mexico [20]. However, in Mexico, few researchers have conducted further research on this topic. There has only been a slow increase in further research on this issue, in particular, and, in general, in English on racism and health in Latin America [11], although relevant research is locally on the rise, as the above mentioned studies from Brazil [9], Mexico [10] and comparatively Brazil, Mexico, Colombia and Peru [11] as well as studies from Chile [21] and Argentina [22] illustrate. In Brazil, for example, discussions regarding institutional racism in the health sector with respect to maternal care have begun to garner more attention [23, 24, 25].

In 2011 and 2012, anthropological research in the state of Veracruz in Mexico was conducted in three medical facilities of the indigenous region Sierra de Totonacapan. The focus was on maternal health in rural Mexico, which inter alia was in the context of the MDGs and a priority within Mexican health policy [26]. As part of a doctoral thesis, the prior rationale to conduct the research was to investigate the effect of such a policy on local reality. However, the aim of this study is a retrospective analysis of the empirical material regarding discrimination and racism within the three medical facilities. The theoretical framework of this study is based on both a human rights approach and on Camara Phyllis Jones’ 2000 published “Levels of Racism” [27]. The author defines and distinguishes racism on three levels: an institutionalized, a personally mediated, and an internalized type of racism.

*Institutionalized racism* is defined as discriminatory access to facilities, goods, services, and opportunities within multi-ethnic societies by race. Consequently, institutionalized racism is evident in the form of commission (action, custom, social practice, law) and in the form of omission. Institutionalized racism manifests in both material conditions and power. *Personally mediated racism* is related to prejudice and discrimination based on race. Personally mediated racism can be intentional or unintentional and manifests due to disrespectfulness, distrust, devaluation, blaming, and dehumanization. *Internalized racism* means the acceptance of racists practiced by individuals affected by any type of racism. The third level of racism refers to self-devaluation, self-rejection, resignation, helplessness, and hopelessness [28].

**Fig 1:**
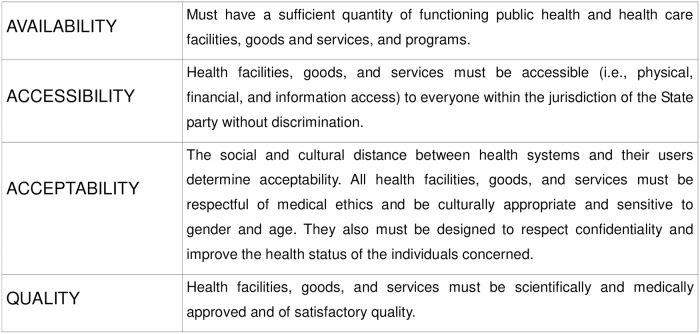
AAAQ framework. Caption credit: CESCR (2000) General comment No. 14. The right to the highest attainable standard of health. Art. 12. Committee on Economic, Social and Cultural Rights. Available from: https://www.refworld.org/pdfid/4538838d0.pdf.

The human right to health, also known as the right to the highest attainable standard of health, comprises legally binding international components. One of the most important components for the right to health is the International Covenant on Economic, Social, and Cultural Rights (CESCR); especially CESCR General Comment No. 14 [26]. The right to health based on General Comment No. 14 encompasses essential elements evaluated by the AAAQ framework of four crucial indicators: availability, accessibility, acceptability, and quality (Fig 1). Availability refers to the existence and quantity of health facilities, goods, and services. Accessibility focuses on the physical and economic access to health facilities goods and services. Furthermore, accessibility has four dimensions: nondiscrimination, physical, economic, and access to information. Acceptability is related to the sensitivity of health facilities, goods, and services to culture and medical ethics. In terms of quality health facilities, goods and services must be scientifically and medically appropriate and of satisfactory quality [29]. Due to this approach, we can address racism as a relevant health concern in Mexico by identifying its *de facto* impact on patients’ health.

## Materials and methods

### Settings

Sierra de Totonacapan is an indigenous region in the highlands of Veracruz in Mexico [30]. Historically and politically, a vulnerable population is concentrated in this marginalized region [31]. The research was conducted in medical facilities in three municipalities of the region. Field research was conducted at the regional hospital of the Sierra de Totonacapan in Entabladero (Hospital de la Comunidad Entabladero) in the municipality Espinal and at local health centers in the municipalities of Coyutla and Filomeno Mata. Ten years before the field research, the regional hospital was built to improve local health care. The location of the field research, namely, the catchment area of the regional hospital, comprised approximately 114,221 residents within its eight municipalities, Espinal, Coyutla and Filomeno Mata are three of them [32].

At the regional hospital, pediatric, internal, and gynecological consultation were offered on different days of the week. In addition to an area for hospitalization, the emergency room and delivery room were the most notable units. Health care services were offered 24 hours per day only at the regional hospital but not at the other facilities. The majority of patients requesting care were pregnant women. Therefore, maternal health care is a priority. A Cesarean section is possible only at the regional hospital but is not available 24 hours per day.

The local health centers in Coyutla and Filomeno Mata offered preventive programs in the form of family planning, vaccination campaigns, and prenatal examinations and general medical consultations and facilities for examinations and deliveries. The duration of the trip from Filomeno Mata to the regional hospital by public transportation or taxi is up to two hours when passing through Coyutla. The duration of the trip to the referral hospitals in the coastal region is even longer. Coyutla is closer to the regional hospital, and the duration is 30 minutes to Entabladero. At night, the only means of transportation to leave the municipalities is a personal vehicle. During the field research, Filomeno Mata offered three consultations in parallel. The opening hours were until late in the evening. At any time, untrained, volunteer interpreters were present to alleviate communication problems between indigenous patients and physicians. In Coyutla, a consultation was offered 5 days per week for eight hours per day. Physicians had experience in basic maternal health care but were not specialized in obstetric care. In addition, the local health center of Filomeno Mata offered health care for normal delivery. In cases of obstetric emergency or risky pregnancy, women were referred to hospitals in the coastal region. Other local health centers such as Coyutla referred pregnant women to the regional hospital in Entabladero for childbirth.

### Study procedures

Methodologically, our field research was based on extended participant observations in Sierra de Totonacapan. The medical facilities of the region offered the opportunity to observe and understand the central values of the local society [33]. For 9 months between 2011 and 2012, the main researcher (Dörr, NM), a German male medical student with experience working in urban Colombian medical facilities and theoretically trained in qualitative research methodology (Universidad Veracruzana, Xalapa/Mexico), maternal health, and global health topics (Justus-Liebig University Giessen/Germany) participated in the work of the regional hospital and local health centers. He was an active and passive member of the medical team, resided directly next to the hospital, and participated in daily life. The research started as a formal internship in the context of the main researchers’ medical carrier, and prior relationships with health professionals and the local population must be interpreted within this framework. Furthermore, the research was conducted in the context of a doctoral thesis [34].

Observations and in-depth interviews were transcribed daily and analyzed in the context of existing empirical material. The starting point of in-depth interviews was the following question: What can be done to improve health care in the region? Based on observations and in-depth interviews, a questionnaire (S1 File) to carry out further in-depth interviews was elaborated. The questionnaire was updated continuously, and its application was dependent on the course of each individual interview. The research was conducted for 4 months in the regional hospital in Entabladero in the municipality of Espinal and further conducted in two local health centers in Coyutla and Filomeno Mata for four more months. During the research in Entabladero and Filomeno Mata, the main researcher lived in the community. While working in Coyutla, he continued to live in neighboring Entabladero.

**Fig 2:**
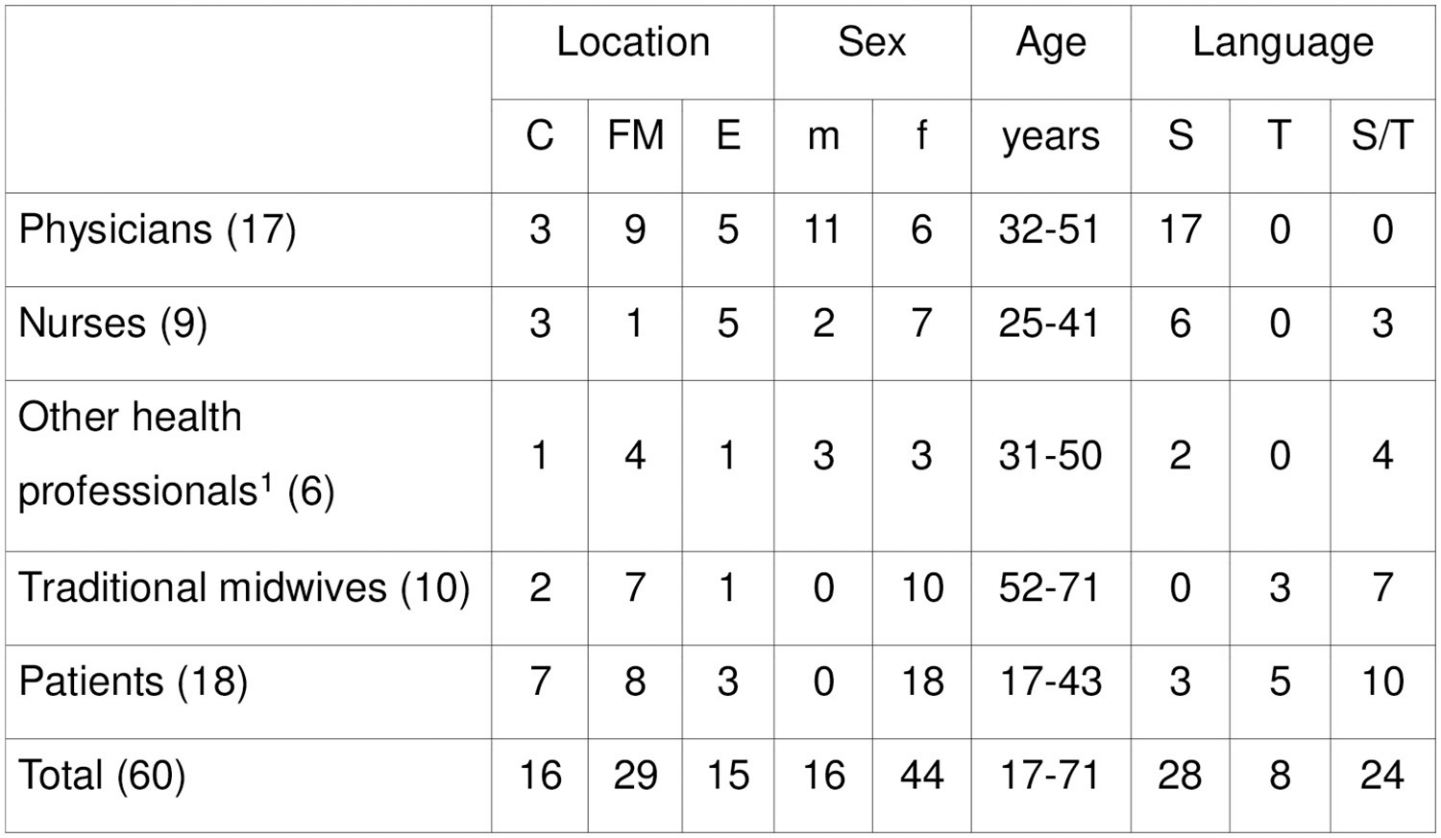
In-depth interview participants. (C) Coyutla. (FM) Filomeno Mata. (E) Espinal. (m) male. (f) female. (S) Spanish speaking. (T) Totonaco speaking. (S/T) bilingual. ^1^ interpreters, apothecaries, social worker and laboratory staff.

The results were based on 60 face-to-face, audio-recorded, in-depth individual interviews (Fig 2). The duration of the interviews was approximately 10 to 60 minutes.

Some informants were interviewed several times to ensure trust and less asymmetric relations. Interviews with health professionals were mainly conducted at the respective medical facility. Patients and midwives were either spontaneously asked for interviews and re-contacted at home or included based on a “snowball sampling” approach [35]. In addition, three focus group discussions with 15 to 18 pregnant women and in each medical facility with local health professionals were performed. The duration of each focus group discussion was approximately 60 minutes. The focus group discussions with pregnant women were conducted in the local health center of Coyutla and focused on the advantages and disadvantages of institutional delivery and discussed the results of the study.

The focus group discussions with health professionals comprised presentations and discussions on the results of the research. Interviews and focus group discussions were conducted in Spanish. In Filomeno Mata, where the majority of the indigenous population speaks only their indigenous language Totonaco, interviews were also conducted in Totonaco. Interviews in Totonaco were conducted with the assistance of a local, female, 18-year-old interpreter and subsequently translated and analyzed in detail in collaboration with her. The interpreter was introduced to the main researcher in the regional hospital. During an informal conversation, in the context of the treatment of a Totonaco speaking relative, in which she also acted as interpreter, she was asked for support to conduct interviews in her hometown of Filomeno Mata. Based on her function as an interpreter and gatekeeper to the indigenous population, we ensured trust between researcher and participants. Furthermore, we discussed the interview implementation and contents with her for further analysis and questionnaire and data consolidation.

### Participants

The patient interviews were conducted with pregnant women and their families who lived in poverty, and some spoke the regional indigenous language Totonaco [36, 37]. The traditional midwives from Coyutla and Filomeno Mata were familiar with the local living conditions or shared them with their patients because they grew up in the region and spoke the indigenous local language as their first language. Within Latin America and especially in Mexico, traditional birth attendance is a relevant concern in contexts of maternal health [38].

The physicians of the medical facilities mainly grew up in the urban centers of the coastal regions Papantla and Poza Rica, where the referral hospitals were located. Some physicians did not reside in but only worked in the rural region. A small part was local and belongs to the nonindigenous, *mestizo* upper class. The physicians did not speak the regional indigenous language, which is a common feature in Mexico [39]. The interviewed physicians were general physicians without intercultural training. Nurses and other professionals, such as interpreters, apothecaries, or laboratory staff, within the medical facilities were partially able to communicate in Totonaco. Many of these individuals grew up in the region and were familiar with local conditions.

The anonymity of the informants was respected without changing the content of the interviews. Apart from a focus group discussion with pregnant women in the local health center of Filomeno Mata, in which the participants refused the interaction, all informants expressed their consent orally to the recording of the collected data. We presumed that the participants were unwilling to express their opinions on pregnancy and childbirth within the local health center in the presence of local staff and the foreign researcher.

Clearance to conduct the study was confirmed by the Department of Health of Veracruz (District No. III Poza Rica), to which the local medical facilities are affiliated. Participants provide verbal informed consent to participate in this study. Written consent was not obtained because of local analphabetism. Dealing with indigenous people, verbal informed consent is a valid culture-specific procedure. Participant consent has been documented via audio recording. Ethical review board wasn’t common practice for anthropological human subject research at Justus-Liebig University Giessen (Germany) by the time field research have been conducted. We protected patient privacy and anonymity, characteristics to prove identity have been deleted immediately.

### Analysis

The continuously collected data analysis was based on grounded theory [40]. Phases of active field research, in the form of participant observation, interviews, and focus groups, were followed by phases of analysis and categorization ("sampling"). The methodological oscillation between data collection and analysis enabled a gradual focus on relevant categories (e.g., culture, family planning, maternal health, traditional midwives, and maternal mortality). The validation of the data was based on methodical triangulation: etic data stemming from observations by the researcher were contrasted with emic data collected by the interviews and reflect the actor’s perspective; both types of data were then contrasted, and their tensions, contradictions, and/or inconsistencies were discussed throughout the focus groups [41]. The methodical triangulation corresponded to the critical application and combination of different research methods by the applied mixed-methods approach. In this manner, categories and their meaning were “thick” and saturated [42]. In other cases, methodological triangulation provided input to new questions and possible understandings. Notably, the main restriction was time. The program ATLAS.ti^®^ was used for archiving and for the qualitative analysis of the data.

## Results

In the following, we highlight the topics of the field research that can be used to identify and discuss the aforementioned levels of racism and other discriminations. All topics are more or less linked to maternal health including reproductive health. We emphasize basic health care within the local health facilities in terms of skilled birth attendants; furthermore, we present results in relation to the concept of culture by referring to the present language barriers and to traditional birth attendants; additionally, we focus on modern contraception, especially female sterilization and intrauterine devices.

### Institutionalized racism

In this section, we present data from the conducted research on how institutionalized racism manifests in material conditions and power relations. In Mexico, indigenous groups represent an extreme context of marginalization and have poor access to medical coverage [20]. The results in this paper confirm this tendency. A nurse from the regional hospital was asked why many pregnant women prefer home births with traditional midwife instead of childbirth in the regional hospital:

*Look! You can summarize the situation: surgeon, gynaecologist, and anaesthetist are the references. But we don’t have them here. Worse still, the births get complicated […]. There isn’t much, we don’t even have infusions. People come and tell us ‘I’ve been badly attended, I’ve been treated badly’. Who knows? That’s how they complain. But we nurses, what can we do if we have nothing? Sometimes there is no doctor. What should I do*?

Particularly, health professionals blame the insufficient human resources, such as specialized health professionals, for poor outcome, and present arguments on availability by expressing the need to have a sufficient quantity and quality of functioning health care goods. An indigenous informant who lived in poverty, stricken Filomeno Mata, explained why people refuse to attend the regional hospital for treatment:

*They want money! Even if they say it’s free, that’s not true. They demand a lot, and then you are also there at the hospital. I think all this is because of the money. Sometimes you are hungry, but you can’t buy anything because you have to pay so much buying food there. We earn very little and even if you have savings, you will spend everything there*.

Thus, people do not leave their municipality because of direct and indirect costs (e.g., accommodation, medical care, food and transport, loss of wages), and this circumstance is crucial when managing pregnancy and birth. This example is a typical example of income-dependent discrimination in health care. Since National Survey on Discrimination 2017, we know low income is related to darker skin color in Mexico [43], so neglect of health care availability and accessibility–in terms of income-dependent discrimination–in indigenous regions are examples of institutionalized racism.

In the context of a focus group discussion in Coyutla, when debating with pregnant women of the region the advantages and disadvantages of the regional hospital, one participant declared the implementation of contraception as a barrier:

*Nowadays, they don’t ask if they want to sterilize you. They don’t ask you if they will do surgery on you. They just do it like this, ‘You will have a surgery because that’s the way it is’. That’s how they tell us there. That’s what some women who were in the hospital tell us. Therefore I don’t want to go there. I really think it’s terrible. At the health center they often told me that I should get sterilization, but I didn’t go*.

In particular, the topic of female sterilization has a deep impact on family planning. Even young indigenous women are regularly confronted with sterilization pressures. Depending on the woman’s age and her number of children, they are sometimes forced to prevent further pregnancies through surgery. This violation of female bodies seems to be based on the belief that indigenous women have no right to make decisions regarding their own bodies, especially if they are supported by the conditional cash transfer programs of the state [44]. They are perceived as anachronistic and child-like by a public health system that equates modernity with the *mestizo* upper class and requires indigenous women to control or even change to promote the modernization of the nation. As a result, medical facilities are generally avoided by the local population when family planning is not conducted in agreement with health professionals.

The strategy of health professionals to increase the coverage of modern contraceptive methods by focusing on female patients can be derived from an incident regarding which we created a memory protocol. The statement of a male doctor corresponds to a conversation with a patient who was about to give birth:

*So, my dear, I’ve heard that you don’t want an IUD [intrauterine device]. Here the hospital policy demands that you have to use it. If you don’t want to, after a month, two months or a year, you have the right to remove the IUD in your local health center. However, you will leave this place with the IUD. These are the rules. If it were up to me, I wouldn’t use it. But the hospital’s policy demands it. I’m just telling, so you don’t feel cheated later*.

In particular, indigenous women are affected by such practices because health professionals assume that they generally reject contraception because of tradition or their sexist husbands, that they are not familiar with modern contraceptive methods, or that they would rather prefer traditional methods. This illustrates a link to the level of personally mediated racism. However, according to Ali et al. *“it is most unlikely that any biological or cultural factor can account for this variability*. *Rather*, *it reflects the policy choices about which methods to promote and biases in family planning services”* [45].

### Personally mediated racism

Through personally mediated racism, interpersonal hierarchies are manifested, which are related to prejudice and discrimination. It can be intentional or unintentional and manifests because of disrespect, distrust, devaluation, blame, and dehumanization.

The language barrier between Spanish speaking professionals and Totonaco speaking patients is often considered problematic, and because of the synonymity of ethnicity, culture, and language by health professionals [46], discrimination due to indigenous language is a common racist practice. An interpreter working at the local health center of Filomeno Mata, asked about culture barriers at the local health center, explained how a doctor treated his patients using insufficient communication; this example shows that patients are discriminated against because they speak an indigenous language, even if resources are available to overcome communication problems:

"*For example, there is a doctor, he doesn’t want any [interpreter] help. He does everything alone. But he doesn’t understand the local dialect. He speaks only Spanish. Who knows how he does it, and then he becomes also aggressive. No wonder, people don’t want to be treated by him. Really! There are some who have stomach pains, others have diarrhea, headaches or back pains, and the doctor, he doesn’t understand them, gives them medication just as he understood. But people aren’t feeling any better*.

The language barrier between parents and children means that children often must mediate between their parents and the health professionals and/or that they must accompany younger relatives to consultations. Due to an increasingly established, so-called “bilingual and intercultural” strategy in pre-primary and primary education, the young population is becoming bilingual, usually speaking the indigenous language within the family and learning Spanish at school and using it afterward in the context of employment. However, as an informant from Filomeno Mata explained, the bilingual or partially Spanish speaking population continues to face discrimination. Notably, communication inside medical facilities is emotionally and substantively demanding comparison with the ordinary use of the Spanish language:

*In fact, the majority in Filomeno Mata doesn’t speak Spanish, that’s the problem! That’s why many people don’t want to go to the hospital. They think they’re being treated badly because of the language. I don’t speak Spanish very well, but I know how to express myself. I know how to help myself when I go to the hospital with my little brother, I know how to answer. But I’m still afraid because I was once treated badly in [the city of] Poza Rica. They insulted me, they insulted me really ugly*.

The local population is often stigmatized by health professionals according to traditions, customs, habits, and culture. Notably, indigenous culture, more than poverty, is declared to be explicitly harmful to health in the following quote from an interviewed male health professional:

*It’s a habit that they believe the midwife more than the doctor. If a pregnant woman has already been attended by a doctor and the doctor tells her to go to the hospital, she does not! They are very isolated by their customs and one of their customs is the midwife*.

According to health professionals, indigenous culture, which was clearly associated with indigenous ethnicity and represented by traditional midwives, leads to disease and the burden of disease for future generations through the mediation of anachronistic world views. Furthermore, culture was held responsible for complex disease processes, perceived insufficient family planning, traditional birth attendants, and high-risk pregnancies or births. In addition, health professionals associated indigenous culture with major health costs and social spending. For example, blaming traditional midwives’ attention (as part of indigenous culture) for maternal mortality or indigenous women for the perceived insufficient family planning are personal-mediated racist practices against indigenous women by the *mestizo* upper class in favor of the nation-state and its modernization [47].

Apparently, personally mediated racism is crucial to the use of contraception and its implementation. The topic illustrates the hierarchy between health professionals and their patients. During a focus group discussion, a woman explained her experiences with family planning in medical facilities:

*In fact, they’re forcing us to sign. They force us to sign the declaration of consent that we want the IUD. If you can’t sign it yourself, the husband has to. I mean that’s a crime. They force you, although you should have the control over your body. If we don’t want, nobody can force us to do something we don’t want. That’s why many women don’t want to go to the hospital for delivery or don’t want to have a doctor take care of them. Because in fact there it’s the doctor who decides and not you*.

However, if ethnic divergence and female sex are part of the interaction, the efforts to limit pregnancies by consequent modern contraceptive methods are even greater. However, undoubtedly, preferences for family planning are subject to health policy and systemic institutionalized racism, which neglects vasectomy as an effective method and reinforces and preserves institutional sexism.

### Internalized racism

Internalized racism means the acceptance of racist practice by individuals exposed to and affected by racism. This level of racism is a methodological challenge. Affected people recognize their ethnicity as harmful, inferior, or worthless. In terms of inferiority, the hegemony of Western medicine over its alternatives is well known in the literature [48]. In general, Western knowledge systems have largely replaced traditional knowledge or forced them to assimilate, such as traditional birth attendants [49]. The first instance is an example of institutionalized racism manifested in material conditions and power relations. However, the following citation demonstrates how a traditional midwife from Filomeno Mata internalized and passed on this inferiority:

*All the doctors, working at the local health center know me. Everything they tell me during the courses, I fulfil. Everything I am told during the courses, I pass on to the women*.

While interviewing traditional midwives, they usually provided assurance that they had fulfilled health professionals’ demands and only implemented what they had learned or been prescribed during official courses. In this context, indigenous midwives and their female patients recognized their culture as harmful, inferior, or even worthless compared with the non indigenous culture and internalized the required changes of their so-called *empirical* practice to semi-skilled attendants [50] and increased referrals to medical facilities, such as the hospital in Entabladero or referral hospitals in the far-away costal region. This abuse of power by the public health system against midwives and patients, in other contexts known as biomedicalization [51], concentrates maternal health within medical facilities to control pregnancy and childbirth. In addition to referrals, mandatory but obsolete and harmful episiotomy [52] during institutional childbirth is an evident example of the biomedicalization of maternal health and common physical violation of female bodies in the Sierra de Totonacapan. Futhermore, biomedicalization is an example of systematic violation of auto-determination and human rights of this indigenous women.

In the following example, a Totonaco speaking female informant explains that she does not like to go to the local health center because of the communication with health professionals:

*I can’t talk to the nurses because I can’t speak Spanish. God forgive me for not having been to school. I’m just being at the health center! They don’t understand me and I don’t understand them either. Basically, we feel exactly the same. That’s quite difficult for me*.

Because of indigenous language, the Totonaco speaking informant had differential access to health care, disease prevention, and health promotion services. In general, the language barrier must be interpreted as co-responsible for progressive indigenous language loss. The rejection of Totonaco native speakers of their language is a result of the successful cultural and educational politics of *indigenismo*, a state-led public policy to assimilate indigenous cultures to Mexican mestizo mainstream culture and to Spanish language usage [53, 54]. During more than half a century, substitutive and transitory bilingual education strategies were used to initially promote literacy and primary education in indigenous languages, but then, in the course of primary education, this instrumental first use of Totonaco was substituted by a Spanish only language policy.

Accordingly, many Totonaco elders are ashamed that they continue to speak their native language while their children or grandchildren embrace Spanish as the “language of modernity.” However, these new generations experience contradictory experiences in school and health institutions: even when they assimilate to the Mexican culture and to the Spanish language, they continue to experience discrimination for being indigenous and perceived as poor. To adapt to this contradiction, many young, educated Totonaco internalize this intersectional mix of racism and aporophobia [55], which the nonindigenous society and institutions impose on them and which they reproduce by identifying with the dominant discourse [56].

## Discussion

Due to our theoretical framework, we acknowledge human rights omissions and violations related to Jones’ Levels of Racism. According to health professionals, there is a relevant omission in the availability of health care goods and human resources. We also showed violations of the right to health in relation to the accessibility of medical facilities, health care, and information by all the aforementioned dimensions: discrimination by economic status, race, gender, ethnicity, language, and consequently, information accessibility. Access to health care without discrimination is one of the most fundamental rights [16]. Mexican anthropologist Menéndez focused on the neglect of discrimination and racism in medical institutions [18]. These factors are forms of institutional violence and abuse of power, which are common racist practices in Mexico in favor of the modernization of the nation-state [47]. This violation is closely associated with the acceptability of health care, medical facilities, and resources. Neglect of the indigenous culture and medical ethics manifests intentionally and unintentionally due to health systems and professionals. A lack of acceptability exists, for example, related to the aforementioned language barrier, traditional birth attendants, or medical ethics regarding the abuse of contraception.

Focusing on the language barrier, health professionals often seem to be doing their indigenous patient a favor by calling an interpreter, but this is a misconception; this act is based on an obligatory legal basis that provides the indigenous patient the right to use their native language in this context. Finally, the lack of skilled birth attendants and prenatal care in terms of scientifically and medically appropriated quality was acknowledged by our research and by the literature [57]. However, by applying a human rights approach, we emphasize racism as a relevant omission and violation of the right to health.

According to our methodology date limitation is given by subjectivity and comprehensibility of qualitative data analysis. Furthermore, our data is limited due to our white male researcher interviewing indigenous female pregnant women. We successfully addressed this problem with the help of our female and indigenous interpreter and due to the extended field work which ensured trust between researcher and participants. This trust have been confirmed when indigenous participants refused comments in the presents of local health professionals during a focus group discussion in Filomeno Mata, while during individual interviews they spoke about intimate contents like family planning. Concurrently, compared to the local doctor-patient relationship, within individual interviews researchers-patient relationship seemed to be less asymmetric. To achieve certain kind of trust the main researcher lived in the community and participated in daily life. Finally, the applied triangulation is one of the methodical strengths of our research.

### Colonial perceptions of health care professionals on indigenous culture

The analysis of special topics shows that lines between the different levels of racism often overlap and reinforce each other. The experience of Totonaco women patients with nonindigenous public health care providers in their region illustrates that health professionals interact with their indigenous patients within an underlying, implicit, but influential framework of enduring colonialism. Our analysis coincides with findings in other indigenous regions of the Americas [58] and beyond [59], which emphasize the continuity and persistence of asymmetrical power relations between public health institutions, on one side, and indigenous communities and their traditional culture of health and healing, on the other side. In these institutionalized contexts, health care professionals tend to reproduce colonially rooted prejudices and stereotypes, whereas their indigenous patients retreat and/or surrender through strategies of avoidance or minimal contact.

These resilient strategies are also observed in colonial and postcolonial state–peasant interactions such as infrastructure projects, agricultural “modernization” measures, and educational and literacy projects, in which “the weapons of the weak” [60] trigger “intimate cultures” of passive and implicit resistance [61], cultures of resistance that follow their own “hidden transcripts” [62] of asymmetrical interaction but do not succeed in developing more symmetrical intercultural relations with the outside world and with state institutions.

To dismantle these types of vicious circles of institutional discrimination, personal stigmatization, and victimized internalization between nonindigenous public health professionals and indigenous patients, the whole institutional system of health care must recognize the institutional racism, that is, colonially rooted means of perceiving, classifying, and simplifying indigenous “otherness.” The insufficient intercultural sensitivity and consciousness on the side of the health care professionals is in sharp contrast with the Totonaco women’s perceptions of ill-treatment, stigmatization, and discrimination they reported in our data. Again, this contrast reflects an ongoing, colonially rooted dichotomy that continues to structure contemporary social and political interactions in Latin America, a dichotomy that has been coined by Santos as the dividing line between memory and amnesia, a line that separates “those who are not able to forget and those who do not want to remember” [63]. The same dividing line has been identified by the Bolivian sociologist Rivera Cusicanqui as the internalized conflicts that continue to define contemporary, often tense, and conflict-ridden indigenous versus nonindigenous interactions [64].

### Intersectionality in health care

Amnesia regarding colonial and postcolonial asymmetries and discriminatory policy traditions such as *indigenismo* prevents health professionals from understanding the historical and contemporary context and from identifying unequal power relations between the Mexican nation-state and Totonaco indigenous communities. At first, segregationist colonialism, and since 1810, assimilationist nationalism, have profoundly shaped the relations between public institutions in Mexico and its indigenous peoples [65]; as a consequence, a strongly racialized “grammar of diversity” [66] has emerged as an underlying cultural logic of intercultural relations between state representatives and communities.

However, our analysis furthermore shows that racism (i.e., in its three aforementioned levels) is not an isolated phenomenon but closely interacts and merges with other, historically rooted sources of discrimination [67, 68]. Our data reveal particularly sexism and misogyny against female patients, who are perceived as ignorant, passive, and irresponsible for their health and that of their children and families. However, sexism is not exclusively personally mediated due to interpersonal or intergender hierarchies, it is manifested by institutionalized sexism-cum-racism. Our results focused on reproductive health as a subject of sexist and racist health policy. Additionally, the Mexican conditional cash transfer program *Oportunidades*, which the majority of the local indigenous population receives, leads to gender-specific and therefore sexist role attributions. The sociologist Molyneux mentioned the retraditionalization of gender roles and identities by the program [69]. In this context, the influence of *Oportunidades* on reproductive health must also be mentioned. Particularly remarkable is the exclusive reference to mothers and their children. Women financially supported by *Oportunidades* are pressured to use certain modern contraceptive methods or to accept medical treatment [43]. For example, doctors who treat women from rural parts of the country who receive *Oportunidades* are more likely to perform a delivery by Cesarean section [70].

This confluence of racism and sexism that we detect in our analysis has also been observed in the case of Afrobrasilian female patients [71] and in the case of Aboriginal women in Canada [72].

Accordingly, diverse identity sources, for example, race and/or ethnicity, gender, age, and class, must be distinguished as different but interrelated and often mutually enforcing sources of discrimination, as well. Only an anti-essentialist definition of these identities will contribute to diversity-sensitive training programs for health professionals. This requires an intersectional approach to the analysis of racism in close relation to sexism, classism, and other ideologies of group asymmetry and supremacy [67, 68].

In the case of the Totonaco communities’ interaction with the Mexican public health system, an intersectional analysis reveals the combined enactment of racist and sexist treatment of indigenous female patients by health professionals that are often linked to other phenomena such as obstetric violence [73, 74]. In these contexts, health professionals reproduce historical amnesia in different but closely interrelated contexts: They do not (want to) remember the colonization of the indigenous body by European health notions, but intertwined with the historical colonization of the female healer by the male health professional and finally the colonization of the rural, “illiterate,” and “poor peasant” body by urban, middle- and upper-class health professionals who perceive otherness in terms of aporophobia [55], hatred, or disgust toward poverty.

### Intercultural competence in health care

Our case study reveals a striking necessity to implement initial and continuous education and training measures for health professionals working in postcolonial frameworks of racial, ethnic, cultural, linguistic, and gender diversity. Intersectional, anti-racist training in intercultural competence is required to prepare health professionals for the development of diversity-sensitive, nondiscriminatory patterns of interaction with their indigenous patients and their families and communities [75]. Intercultural competence is mostly defined as the ability to communicate, perform, and interact as a professional in diverse and heterogeneous settings with people and groups from different cultural or subcultural, social class, ethnic, national, religious contexts; different age groups and/or gender roles; and different abilities. These abilities are manifested in the health professional´s daily work through their capacity to overcome the ethnocentrism from their group of origin, conduct “code switching” to shift their perception of their patients’ perceptions, notice and identify their positionality and relationality, and make cultural and linguistic “translations” between different normative and cultural systems, including different systems and logics related to health, illness, healing, and well-being [71].

These types of experiences require knowledge and know-how that—unlike culture-specific competencies—are not limited to a specific culture but that refer to the relations among different cultures. In the last decades, in educational research and in linguistics and communication, these skills have been called competences. These fundamentally linguistic and communicative competences are identified and explained in didactic sequences that help the apprentice reflect on their encounter process with the “other” regarding cultural differences and consciously thematize them, to trigger intercultural learning processes that avoid the frequent misunderstandings and misperceptions in these types of encounters. The “intercultural speaker” [76] can observe from the perspective of their counterpart, of handling insecurities or ambivalences product of the cultural difference to finally develop an empathic comprehension of the other’s perspective.

Analytically, Byram distinguished six types of communicative competences: linguistic competences (the ability to generate sentences with meaning in the target language), sociolinguistic competences (the ability to identify structures and relationships that influence the choice of languages), discursive abilities (the ability to create and understand a text with the appropriate strategies), strategic competences (the ability to cope with misunderstandings and mistakes within the communication of a foreign, non-native language), sociocultural competences (the ability to recognize the sociocultural context in which a language is spoken), and social competences (the ability to interact with others) [76].

The second most important approach in the field of intercultural competences, in this case, inspired mostly by psychology rather than linguistics, is the Development Model of Intercultural Sensibility, developed by Bennett. His model explains the social construction of cultural differences and how to experience these cultural differences in a more sensitive, complex, and nonbiased manner. To achieve this capacity, individuals transition through six stages, in which the cultural differences are formed. The six stages comprise three "ethnocentric" stages and three "ethnorelative” stages. First, individuals experience the ethnocentric stages: denial of differences, defensive attitude toward differences, and minimization of differences. Subsequently, individuals can access the ethnorelative stages: acceptance of difference, adaptation to difference, and integration of an intercultural world view. These stages have been applied to the counseling, training, and intercultural training of professionals that have been “exposed” to cultural differences and diversities because they allow the identification of different levels of intercultural competences and help reverse biases and gaps regarding incompetence and insufficient sensitivity [77, 78].

## Conclusion

Racism, and other related ideologies such as sexism and classism, are neglected but relevant health concerns in Mexico. Our data demonstrated, despite the study’s limitations (i.e., a lack of statistical representativity, one indigenous region, and a small sample), how institutionalized racism manifests in the lack of skilled human resources, shortage of functioning health care goods in terms of quantity and quality, and income-dependent discrimination within local medical facilities. Family planning and contraception are examples of the confluence of racism and sexism regarding an intersectional approach. Personally mediated racism manifests in discrimination and stigmatization due to language, traditions, customs, habits, and culture. We also highlighted examples of internalization by victims of racism and reproduction of discrimination by those. According to the AAAQ indicators, racism violates the right to health in all dimensions.

According to Jones’ theoretical framework, in the Sierra de Totonacapan, the indigenous people and their health care are affected by all the levels of racism. Furthermore, our data demonstrated a link between those levels, and the relationship between the three levels of racism that Jones called “a Gardener’s Tale.” A gardener who has two flower boxes with different types of soil. The story illustrates notable aspects of institutionalized racism. *“There is the initial historical insult of separating the seed into the two different types of soil; the contemporary structural factors of the flower boxes*, *which keep the soils separate; and the acts of omission in not addressing the differences between the soils over the years*.*”* At the end of her metaphor, she concludes by pointing out the fundamental impact of institutionalized racism [27].

We discussed enduring colonialism, which emphasizes the continuity and persistence of asymmetrical power relations in health care due to health disparities by race, gender, culture, and ethnicity. In the context of intercultural medicine, the relevance of intercultural competence and empowerment to manage personally mediated and internalized racism is understood [79]. However, when focusing on maternal health, the impact of skilled birth attendants and its link to social and structural determinants had been well investigated [57, 80]. Regarding the applied framework of racism, to manage all three levels of racism in an intersectional manner, anti-racist training in intercultural competence is required to prepare health professionals to develop diversity-sensitive, nondiscriminatory patterns of interaction with their indigenous patients and their families and communities. This training does not substitute existing models of public health care but aims to interculturalize them to increase health professionals’ sensitivity to racism and sexism as obstacles to providing culturally adequate health services in contexts of cultural diversity and colonial-origin asymmetries. To dismantle the heritage of colonialism and solve the contemporary omissions and violations of persistent health disparities due to racism, Mexico requires public investment in functioning health goods, skilled birth attendants, and cultural competence to address the availability, accessibility, acceptability, and quality of (indigenous) health care.

## Supporting information

S1 File Questionnaire of in-depth interviews. (DOC)

S2 File ISSM_COREQ_Checklist. (PDF)
