# Supplementary material for: Racism against Totonaco women in Veracruz: Intercultural competences for health professionals are necessary
Source: PLoS One. 2020 Jan 14;15(1):e0227149. doi: 10.1371/journal.pone.0227149 (PMC6959590; doi:10.1371/journal.pone.0227149)
Supplement: S1 File — (DOC) [file pone.0227149.s001.doc]

# Questionnaire of in-depth interviews

These questions were developed during the research process. Normally, and dependent on the discourse of the interview, not all questions were asked in all interviews. In some cases, the content of the questions was modified or specified to adapted to the individual interview situation.

**Questions to traditional midwives:**

What can be done to improve health care in the region?

How long have you worked as a midwife?

How did you start working as a midwife?

How did you learn how to be a midwife?

What is a traditional midwife?

How much money do you charge?

How many patients do you have at the moment?

Which patients come to you?

What must a patient consider before, during, or after childbirth?

Do you work together with the local health center?

What are the differences between doctors and midwives?

Do patients prefer to go to the regional hospital or local health center?

What fears do your patients have?

Why do pregnant women prefer not to go to the regional hospital?

How do you evaluate doctors' work?

Why do you refer your patients to the regional hospital or local health center?

When did doctors begin to participate in midwifery training?

What has changed as a result of midwifery training?

How would you rate your midwifery training?

How do your patients evaluate midwifery training?

How can an individual imagine childbirth with a midwife?

Do your patients attend antenatal care?

Why are women told to have fewer children?

Have women died during pregnancy or childbirth in the municipality?

What are the causes of maternal mortality?

How can the death of a pregnant woman be prevented?

What roles does the husband play in pregnancy and childbirth?

What do you think about family planning?

**Questions to health professionals:**
What are the relevant concerns at the regional hospital?

What can be done to improve health care in the region?
What impact does the regional hospital have?
What role does the culture of the patient have?
What are the main barriers between the regional hospital and local health center and the local population?
Do communication problems affect health care?
Are there differences in care between indigenous and nonindigenous patients?
How do you perceive the work of traditional midwives?
What is your opinion of traditional medicine?
What makes a good midwife?
How can I imagine cooperation with traditional midwives?
Why do many women prefer a traditional midwife?
What has changed since the *seguro popular de salud[[1]](#footnote-2)*?
How do you evaluate the *Oportunidades[[2]](#footnote-3)* program?
Why are many women worried about going to the regional hospital?
How high is the maternal mortality rate in the region?

Why do patients die during pregnancy and childbirth?
How can the maternal mortality rate be reduced?
When did maternal mortality begin to be a priority?
Are there relationships between maternal mortality and traditional midwives?

How are midwives encouraged to join training?
What problems do you associate with family planning?
Which paper does the male population play for family planning?
Why are patients afraid of surgery?
How is OTB [Oclusión Tubaria Bilateral] introduced to female patients?
Why do all women receive the DIU [Dispositivo Intrauterino] after childbirth?

**Questions to patients:**

What can be done to improve health care in the region?

How do you perceive health care in the regional hospital or local health center?

Have you ever gone to the regional hospital or local health center to give birth?

What experience do you have with the regional hospital or local health center?

Do you receive prenatal examinations?

Why would you go to a midwife or a local health center for delivery?

Do you feel forced to go to the regional hospital or local health center because of Oportunidades?

What are the barriers to meeting a midwife or at the local health center?

Do you are nervous about giving birth?

According to which criteria did you choose your midwife?

Are midwives a tradition?

What are the differences between midwives and doctors?

What does a woman have to consider before, during, and after pregnancy?

What are the causes of child death?

What are the causes of maternal death?

Have they been told about the operation to avoid having children?

What do they tell you about OTB?

What do you think about surgery?

What does your husband think about surgery?

1. The *seguro popular de salud* is a financing instrument launched by the Mexican Ministry of Health in 2003. Under the leadership of the Comisión Nacional de Protección Social en Salud, the *seguro popular de salud* secures health care for the uninsured population. [↑](#footnote-ref-2)
2. *Oportunidades* is a Mexican Conditional-Cash-Transfer Program launched 1997. [↑](#footnote-ref-3)
